# IUC26529-89 MERIT AWARD: Sex Differences in Enfortumab Vedotin Efficacy and NECTIN-4 Levels in Metastatic Urothelial Cancer

**DOI:** 10.1093/oncolo/oyag286.002

**Published:** 2026-08-21

**Authors:** T Taha, A Alpert, A Rizzo, F Ciccimarra, J Bellmunt, S Gupta, R Kanesvaran, Y Urun, M Santoni, I Waldhorn

**Affiliations:** The Royal Marsden NHS Foundation Trust, London, UK; Division of Oncology, Rambam Health Care Campus, Haifa, Israel; Oncology institute, Tzafon Medical Center, Poriya, affiliated with Azrieli Facu - United Kingdom; Division of Oncology, Rambam Health Care Campus, Haifa, Israel - Israel; S.S.D. C.O.R.O. Bed Management Presa in Carico, TDM, IRCCS Istituto Tumori “Giovanni Paolo II”, Viale Orazio Flacco 65, 70124, Bari, Italy - Italy; S.S.D. C.O.R.O. Bed Management Presa in Carico, TDM, IRCCS Istituto Tumori “Giovanni Paolo II”, Viale Orazio Flacco 65, 70124, Bari, Italy - Italy; Dana Farber Cancer Institute, Harvard Medical School, Boston, MA, USA - United States; Taussig Cancer Institute, Cleveland Clinic, Cleveland, OH, USA - United States; Division of Medical Oncology, National Cancer Centre Singapore, Singapore - Singapore; Department of Medical Oncology, Ankara University Faculty of Medicine, 06620 Ankara, Turkey - Turkey; Oncology Unit, Macerata Hospital, via Santa Lucia 2, 62100, Macerata, Italy - Italy; Division of Oncology, Rambam Health Care Campus, Haifa, Israel - Israel

## Abstract

**Background:**

Antibody-drug conjugates (ADCs) represent an emerging class of anti-cancer therapies. Enfortumab vedotin (EV), a NECTIN-4–targeting ADC, has recently been integrated into the treatment landscape for urothelial cancer (UC). Prior subgroup analyses suggest sex may influence treatment efficacy – a factor often underexplored in research. To investigate this, we conducted a multi-center real-world data analysis, complemented by translational research, to gain deeper insight into this disparity.

**Methods:**

Clinical outcomes were collected from the ARON2-EV retrospective study. Kaplan–Meier and log-rank tests were used for analysis. TCGA data for patients diagnosed with T2-T4a UC were analyzed for NECTIN-4 expression, sex differences, smoking status, molecular subtypes and regulating pathways.

**Results:**

454 patients with metastatic UC treated with EV across 51 centers in 18 countries were included. Overall survival was 13.6 months in males vs 7.7 months in females (p<.001), consistent after stratification by ECOG status, tumor histology, smoking status and prior therapy. Males also had longer time on-treatment (13.3 vs 7.4 months, p<.001). Objective response rate was similar (45% in males vs 36% in females), but primary refractory rates differed significantly (26% vs 44%, respectively, p = .008). NECTIN-4 expression was higher in men compared to women (p<.001), an effect that was augmented in smokers. Women were more often diagnosed with basal histology, which exhibits lower NECTIN-4 expression; However, differences persisted even after stratification by molecular subtype (p = .002). NECTIN-4 expression was most strongly associated with estrogen response gene set (p<.001), and smoking was found to reduce estrogen levels specifically in women.

**Conclusions:**

Females appear to benefit less from EV treatment, potentially due to lower Nectin-4 expression driven by molecular subtype, mutational signatures, and the differential impact of smoking on estrogen levels. As ADCs continue to emerge as a novel class of therapy, these findings of an ADC with sex differences underscore the importance of evaluating the influence of sex on ADC efficacy and toxicity in clinical trials.

**Acknowledgements:**

ARON Research Foundation ETS for its support and encouragement. All other co-authors: Jalal Baranseh, Enrique Grande, Sebastiano Buti, Francesco Massari, Fernando Sabino Marques Monteiro, Giandomenico Roviello, Cristian Lolli, Hideki Takeshita, Avivit Peer, Aristotelis Bamias.

